# Influence of Intraocular Pressure on Clinical Decision-Making in Glaucoma Management

**DOI:** 10.1001/jamaophthalmol.2025.5593

**Published:** 2026-01-08

**Authors:** Ashley Polski, Ben J. Brintz, Rachel Hess, Kensaku Kawamoto, Felipe A. Medeiros, Joshua D. Stein, Brian C. Stagg

**Affiliations:** 1 Moran Eye Center, Department of Ophthalmology, University of Utah, Salt Lake City; 2 Division of Epidemiology, Department of Internal Medicine, University of Utah, Salt Lake City; 3 Division of Population Health Sciences, Department of Internal Medicine, University of Utah, Salt Lake City; 4 Department of Biomedical Informatics, University of Utah, Salt Lake City; 5 Bascom Palmer Eye Institute, Department of Ophthalmology, University of Miami, Miami, Florida; 6 Kellogg Eye Center, Department of Ophthalmology, University of Michigan, Ann Arbor

## Abstract

**Importance:**

Understanding of intraocular pressure (IOP) as a continuous risk factor for glaucoma has evolved over time, and IOP reduction is widely acknowledged as the mainstay of treatment. However, the impact of specific IOP levels on clinical decision-making remains an underexplored topic.

**Objective:**

To assess how IOP levels influence the decision to initiate or escalate glaucoma therapy in clinical practice.

**Design, Setting, and Participants:**

In this retrospective, multicenter cohort study, the Sight Outcomes Research Collaborative (SOURCE) ophthalmology data repository was used to identify clinic encounters between October 2009 and January 2022 for patients with glaucoma with IOPs ranging from 12 mm Hg to 25 mm Hg. Data analysis was performed from July 2024 to September 2025.

**Main Outcomes and Measures:**

The primary outcome was whether IOP-lowering therapy was initiated or escalated after each clinic encounter, defined as a new prescription for IOP-lowering medication within 1 week of the encounter, laser treatment within 4 weeks, or glaucoma surgery within 8 weeks. The rate of treatment initiation at different IOP levels was measured, and then mixed-effects logistic regression was used to model the odds of treatment initiation at specific indicator IOP levels.

**Results:**

This analysis included 1 866 801 clinic encounters from 184 504 eyes of 94 232 unique patients across 7 sites in SOURCE. Mean (SD) patient age was 69.5 (10.8) years, and of the total clinic encounters, 1 084 827 (58.1%) included female patients. The rate of IOP-lowering treatment increased with higher IOP levels, with the largest acceleration in treatment rate at IOPs of 22 mm Hg or higher. With mixed-effects logistic regression modeling, an indicator IOP of 22 mm Hg had a greater effect on treatment initiation (odds ratio, 1.11; 95% CI, 1.08-1.14) compared with lower indicator IOPs.

**Conclusions and Relevance:**

In this cohort study, while clinicians seem to generally use IOP as a continuous risk factor in their treatment patterns, with higher rates of glaucoma therapy at increasing IOP levels, these findings suggest that the historical IOP cutoff of 22 mm Hg may still influence clinician decision-making in glaucoma management. Improved clinical decision support may be useful to assist clinicians with using IOP as a continuous risk factor in their decision-making.

## Key Points

**Question:**

How do intraocular pressure (IOP) levels influence clinical decision-making in contemporary glaucoma management?

**Findings:**

In this cohort study, clinicians across 7 US academic ophthalmology institutions demonstrated higher glaucoma treatment rates at increasing IOP levels but were disproportionately more likely to initiate IOP-lowering treatment for IOPs of 22 mm Hg or higher than for IOPs less than 22 mm Hg.

**Meaning:**

While clinicians generally use IOP as a continuous risk factor in their glaucoma treatment patterns, the historical definition of normal IOP being less than 22 mm Hg may still influence clinician decision-making in glaucoma management.

## Teaser

This cohort study using data from the Sight Outcomes Research Collaborative (SOURCE) data repository assesses how intraocular pressure levels influence the decision to initiate or escalate glaucoma therapy in clinical practice.

## Introduction

Glaucoma is a type of optic neuropathy characterized by progressive visual field loss and damage to the optic nerve that is often, but not always, associated with elevated intraocular pressure (IOP).^1^ While numerous factors influence glaucoma risk—including age, IOP, family history, and corneal thickness—IOP is the primary modifiable risk factor for glaucoma progression.^2,3,4^ The exact mechanisms linking elevated IOP to glaucomatous damage are not fully understood; however, IOP reduction has consistently been shown to slow disease progression and preserve visual function.^5,6,7^ As such, lowering IOP with medication, laser treatment, and/or surgery remains the cornerstone of glaucoma management.^1^

In recent decades, understanding of the association between IOP and glaucoma has evolved significantly. Landmark studies, such as the Ocular Hypertension Treatment Study,^3^ the Early Manifest Glaucoma Trial,^8^ and the Advanced Glaucoma Intervention Study,^9^ have all demonstrated the importance of IOP-lowering treatment to minimize glaucomatous progression. The Collaborative Normal-Tension Glaucoma Study^7^ similarly showed that IOP reduction is crucial for slowing visual field progression, even for patients with glaucoma with low or physiologic IOP levels. Current glaucoma preferred practice guidelines reflect these findings, recommending a reduction of IOP by at least 25% compared with pretreatment levels.^10^ Few smaller studies have assessed glaucoma treatment patterns relative to IOP, showing that patients with elevated IOP are generally more likely to undergo treatment escalation than patients with lower IOP.^11,12^ However, the impact of specific IOP levels on clinician decision-making to initiate or escalate glaucoma therapy remains an underexplored topic.

In this study, we aimed to address this gap by evaluating how IOP levels influence the decision to initiate or escalate IOP-lowering therapy in patients with glaucoma. Using a large, multicenter data repository, we examined treatment patterns from academic eye centers across the US to better understand how IOP measurements guide decision-making in contemporary glaucoma care.

## Methods

This study was approved by the University of Utah institutional review board. Informed consent was waived, given that patient data were deidentified. The research adhered to the tenets of the Declaration of Helsinki and complied with the Health Insurance Portability and Accountability Act. The Strengthening the Reporting of Observational Studies in Epidemiology (STROBE) reporting guidelines were followed.

### Data Source

In this cohort study, we retrospectively reviewed electronic health record data from the Sight Outcomes Research Collaborative (SOURCE) ophthalmology data repository between October 2009 and January 2022. This large, multicenter repository contains deidentified data from all patients receiving eye care at participating academic medical centers within the research consortium. Although the data in SOURCE are deidentified, patients are assigned privacy-preserving tokens from Datavant, Inc, allowing longitudinal follow-up over time. Information captured within the SOURCE repository includes patient demographics, diagnoses based on *International Classification of Diseases Ninth Revision (ICD-9) *and *Tenth Revision (ICD-10)* billing codes, procedures based on *Current Procedural Terminology* (*CPT*) codes, and both structured and unstructured data from all clinical encounters (including clinic visits and surgeries).^13,14,15,16,17^

### Study Design

We identified all clinic encounters within the SOURCE repository for patients with a documented diagnosis of glaucoma (based on *ICD-9* codes 365.X and *ICD-10* codes H40.X) in at least 3 separate clinic encounters and with IOP measurements ranging from 12 to 25 mm Hg in at least 1 eye (based on measurements with any tonometry instrument, as the SOURCE database does not consistently specify the type of tonometry device used). For any encounters that included multiple IOP measurements of the same eye, the highest IOP value was used. The main outcome measure was the initiation of IOP-lowering treatment after an encounter, which was defined as (1) a new prescription order for a type of IOP-lowering medication not previously prescribed for the patient during their time in the database within 1 week of an encounter, (2) a *CPT* billing code for selective laser trabeculoplasty within 4 weeks, or (3) a *CPT* billing code for a glaucoma surgery within 8 weeks (eTable 1 in Supplement 1). These time cutoffs were determined and agreed upon by fellowship-trained glaucoma specialists from 3 separate academic eye centers based on more than 5 years of experience treating patients with glaucoma. Treatment initiation included starting treatment in previously untreated eyes or the escalation of treatment in previously treated eyes.

### Statistical Analysis

To evaluate the association between IOP and glaucoma treatment in our study cohort, we plotted the rate of treatment initiation at each IOP level between 12 and 25 mm Hg. An apparent inflection point in treatment initiation was noted above an IOP of 21 mm Hg, which was further assessed using piecewise logistic regression modeling (see eTable 2 in Supplement 1). Mixed-effects logistic regression was then used to model the probability of IOP-lowering treatment based on a smaller subset of IOP levels. Each model included a continuous IOP variable followed by an indicator IOP value—17, 18, 19, 20, 21, 22, 23, 24, or 25 mm Hg—giving a total of 9 models. For each of the 9 models, the continuous IOP variable included a range of IOPs from 12 mm Hg up to the model-specific indicator (for example, the model with an indicator IOP of 20 mm Hg included IOP measurements between 12 and 20 mm Hg). The purpose of these models was to identify an inflection point in treatment probability by assessing the magnitude of change from the trend that could be attributed to the highest IOP level included in the model (the indicator IOP). While these IOP and treatment data are nuanced and nonlinear, a high-order polynomial fit does not infer where an inflection point occurred, and therefore, change point detection was used to directly assess the change from the general trend at each additional point added in the analysis. In other words, if the indicator IOP demonstrated a disproportionate increase in treatment probability compared with the continuous IOP values before it, this would suggest an inflection point in treatment rate at that indicator IOP level. All models accounted for patient age and number of clinic visits, and random effects for visit clinician and patient eye were also included to account for within-clinician and within-eye correlation over time. The binary outcome variable for each model was 0 (no treatment change) vs 1 (initiation or escalation of IOP-lowering treatment). The likelihood of treatment (represented by an odds ratio [OR] as a function of IOP) was determined for each of the 4 indicator IOP values. A subanalysis was also performed using the same mixed-effects logistic regression modeling and 9 indicator IOPs, but only encounters associated with treatment initiation (in the setting of no prior treatment for a >1-year look-back period) were included. *P* values were 2-sided, and *P* < .05 was considered significant. R version 4.5.1 (R Foundation) was used for analyses.

## Results

### Study Cohort and Demographics

**Figure 1.**
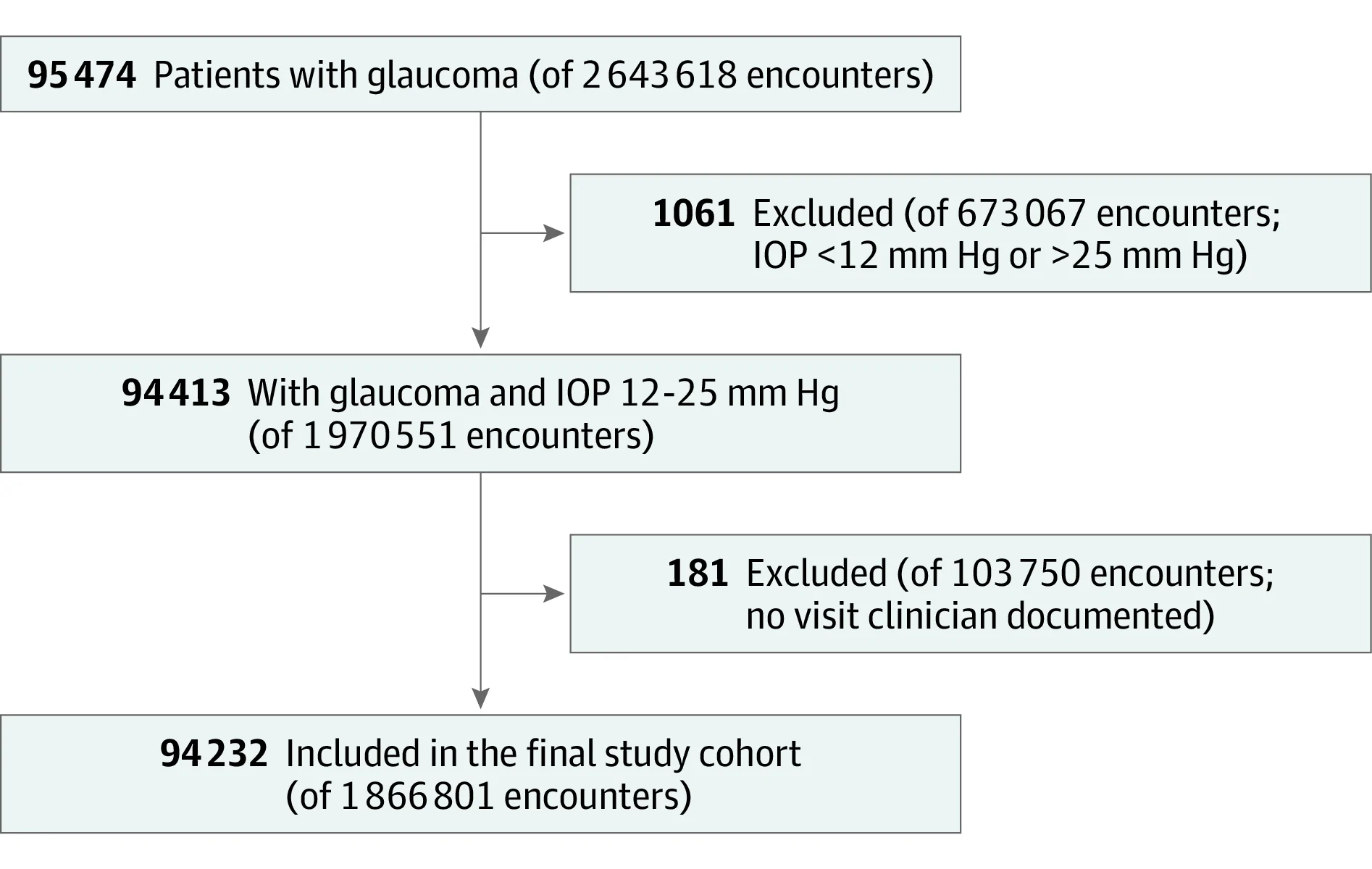
Study Attrition Diagram of Eligible Patients With a Documented History of Glaucoma and Intraocular Pressure (IOP) Measurements Ranging From 12 to 25 mm Hg

Of 7 academic eye centers within the SOURCE consortium, a total of 1 866 801 clinic encounters were included from 184 504 eyes of 94 232 unique patients (Figure 1). Mean (SD) patient age was 69.5 (10.8) years, and of the total clinic encounters, 1 084 827 (58.1%) included female patients.

### Association Between IOP Level and Treatment Initiation

**Figure 2.**
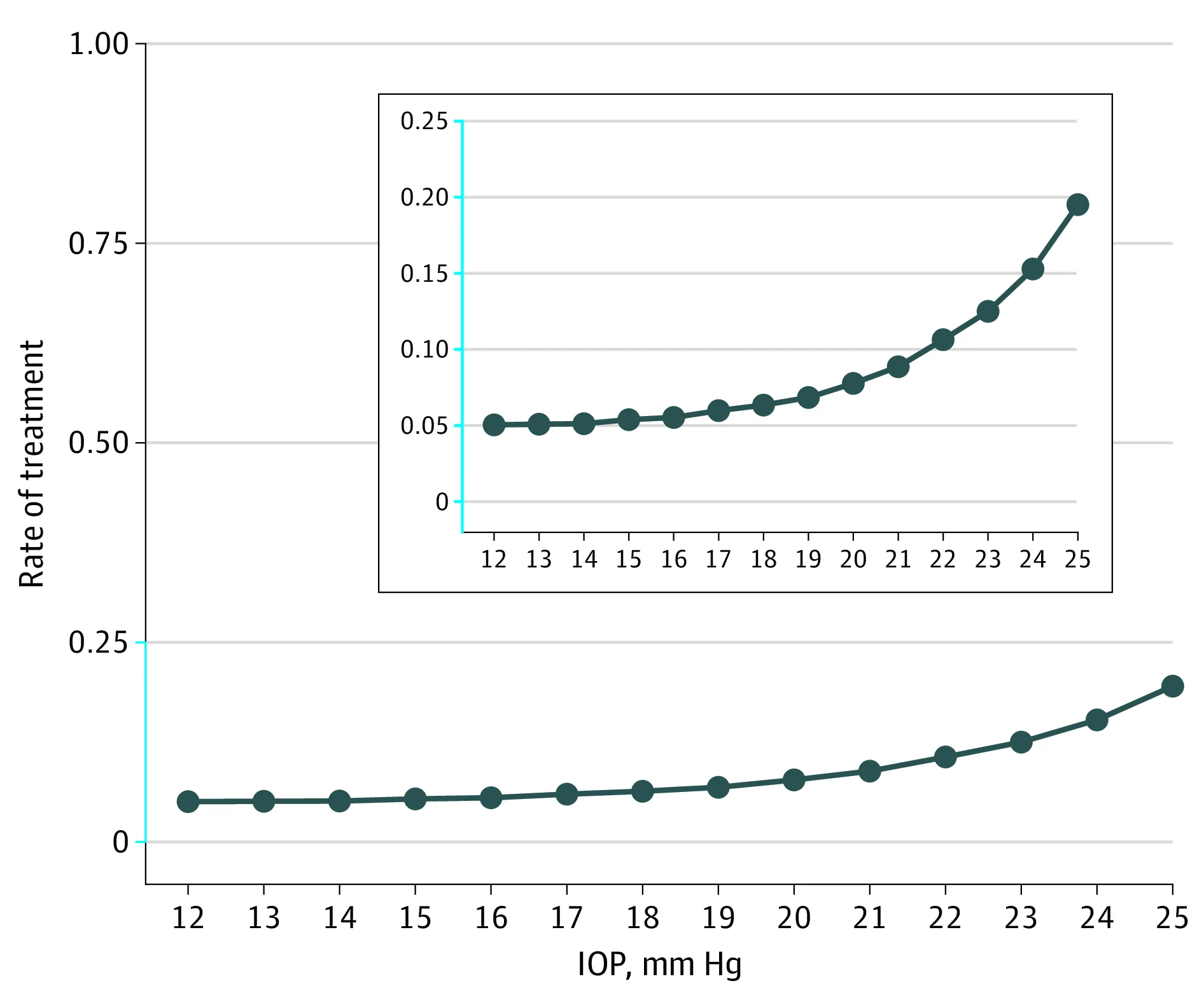
Rate of Treatment Initiation Based on Intraocular Pressure (IOP) Level Treatment rates appeared to accelerate at IOPs >21 mm Hg.

**Figure 3.**
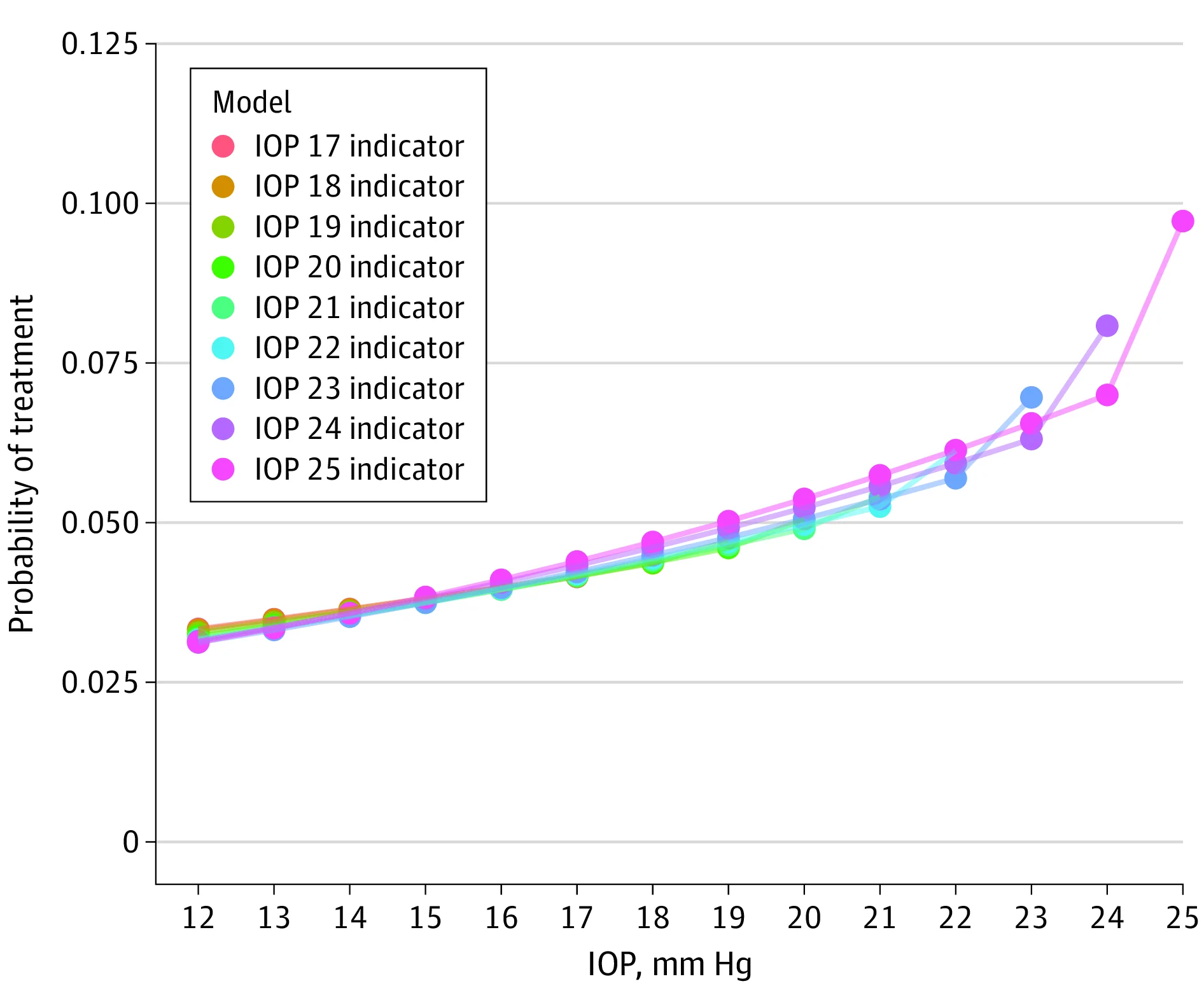
Mixed-Effects Logistic Regression Models Demonstrating the Probability of Treatment Initiation at Increasing Intraocular Pressure (IOP) Levels Each of the models showed similar trends in treatment probability at lower IOP values. However, the increase in treatment probability was greater at the indicator IOP of 22 mm Hg compared with prior values, and treatment probability continued to accelerate at values above the indicator IOP of 22 mm Hg. Note: the y-axis (probability of treatment) only shows values between 0 and 0.125 and is therefore a magnified view of the data points.

**Figure 4.**
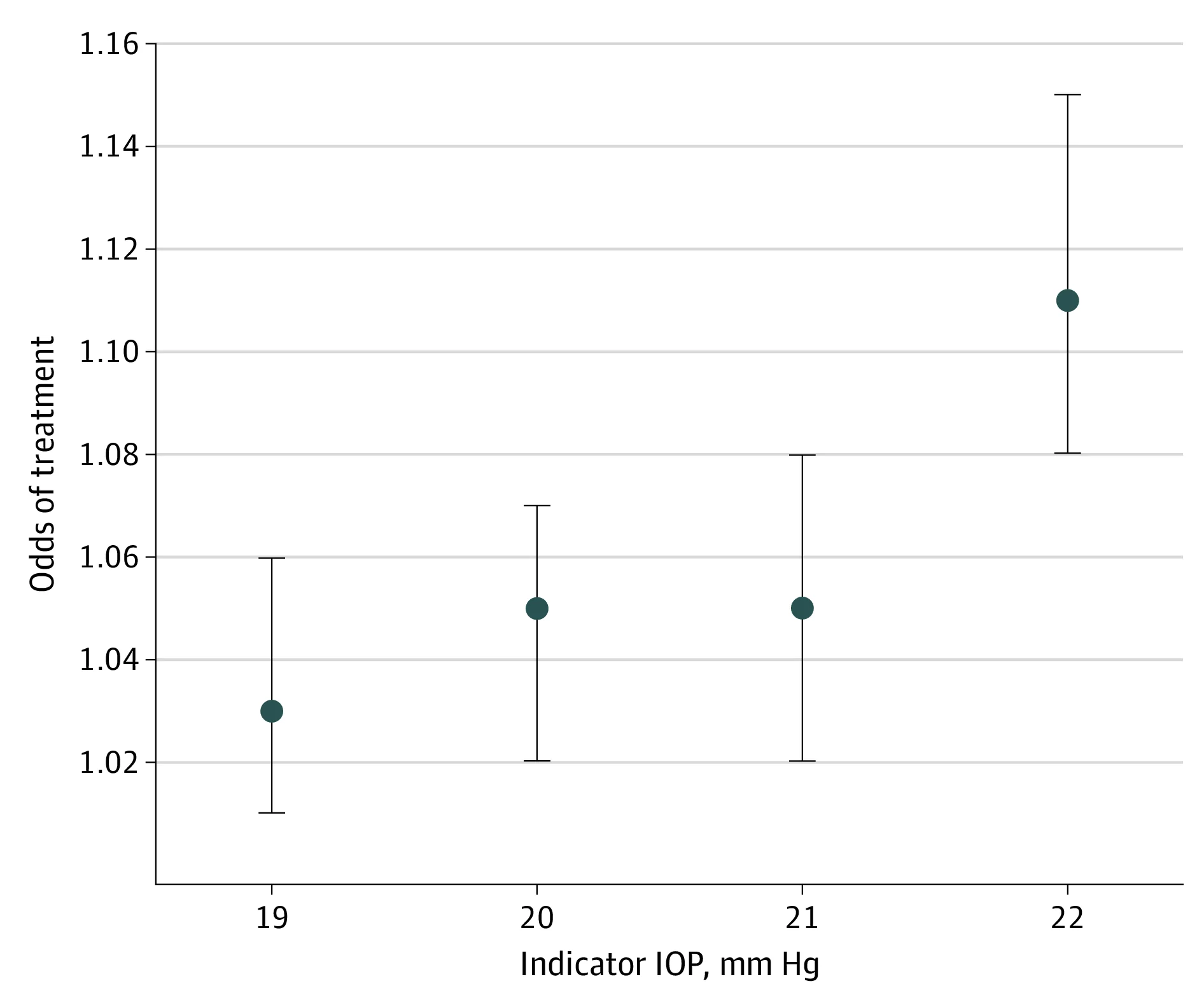
Odds Ratios (With 95% CIs) of Treatment Initiation at Indicator Intraocular Pressures (IOPs) of 19, 20, 21, and 22 mm Hg The odds of treatment initiation were higher at an IOP of 22 mm Hg compared with indicator IOPs of 19, 20, and 21 mm Hg. Error bars represent 95% confidence intervals.

Treatment was initiated after 248 349 encounters (232 372 [93.6%] with medication, 9259 [3.7%] with laser treatment, and 6718 [2.7%] with surgery). For IOPs between 12 and 25 mm Hg, the rate of glaucoma treatment initiation progressively increased with increasing IOP levels. Qualitatively, treatment rate appeared to accelerate at IOPs of 22 mm Hg or higher (Figure 2). With piecewise logistic regression modeling, a larger increase in logistic regression slope was also noted at an indicator IOP of 22 mm Hg compared with indicator IOPs of 19, 20, or 21 mm Hg (eTable 2 in Supplement 1). The 9 mixed-effects logistic regression models each demonstrated similar trends in treatment probability at lower IOP values, with treatment being gradually more likely at increasing IOPs. However, there was a larger increase in the odds of treatment at the indicator IOP of 22 mm Hg relative to the continuous IOP values below it (Figure 3). For both treatment initiation and escalation combined, indicator IOP values of 19, 20, and 21 mm Hg resulted in treatment odds between 1.03 and 1.05 (19 mm Hg: 1.03; 95% CI, 1.01-1.06; 20 mm Hg: 1.05; 95% CI, 1.02-1.07]; and 21 mm Hg: 1.05; 95% CI, 1.02-1.08). At the indicator IOP of 22 mm Hg, treatment odds increased to 1.11 (95% CI, 1.08-1.14; *P* < .001) (Figure 4; Table). When only treatment initiation was included, a similar pattern was demonstrated. Indicator IOP values of 19, 20, and 21 mm Hg resulted in treatment odds between 1.03 and 1.12 (19 mm Hg: 1.03; 95% CI, 0.99-1.08; 20 mm Hg: 1.06; 95% CI, 1.02-1.10; and 21 mm Hg: 1.12; 95% CI, 1.07-1.16), whereas an indicator IOP of 22 mm Hg was associated with an increase in treatment odds to 1.23 (95% CI, 1.18-1.29; *P* < .001). (See the Table for the treatment odds from all 9 mixed-effects logistic regression models.)

**Table.** Mixed-Effects Logistic Regression Models From 184 504 Eyes, Demonstrating Odds of (1) Treatment Initiation and Escalation and (2) Treatment Initiation Only at Increasing Intraocular Pressure (IOP) Levels

Indicator IOP, mm Hg	OR (95% CI)^a^	
	Treatment initiation or escalation	Treatment initiation only
17	0.9955 (0.9734-1.0180)	1.0033 (0.9654-1.0428)
18	1.0080 (0.9865-1.0299)	1.0085 (0.9722-1.0462)
19	1.0316 (1.0078-1.0559)	1.0334 (0.9935-1.0750)
20	1.0453 (1.0214-1.0698)	1.0594 (1.0194-1.1010)
21	1.0477 (1.0204-1.0757)	1.1158 (1.0692-1.1644)
22	1.1110 (1.0809-1.1420)	1.2314 (1.1792-1.2859)
23	1.1638 (1.1260-1.2028)	1.3091 (1.2448-1.3768)
24	1.2222 (1.1825-1.2634)	1.4478 (1.3779-1.5212)
25	1.3334 (1.2814-1.3875)	1.5762 (1.4871-1.6707)

Abbreviation: OR, odds ratio.

^a^ In both subgroups, treatment probability increased gradually at increasing indicator IOPs, and the first instance of a significant increase in treatment odds with nonoverlapping 95% confidence intervals was at an indicator IOP of 22 mm Hg.

## Discussion

In this study, we evaluated a large, multicenter ophthalmology data repository to assess how IOP levels influence the decision to initiate or escalate glaucoma therapy in clinical practice. Historically, clinicians were taught the commonly held belief that IOPs of 22 mm Hg or higher were abnormal and warranted treatment for glaucoma.^18^ This dichotomization between so-called abnormal (≥22 mm Hg) and normal (<22 mm Hg) IOP was first derived from a large 1958 study based in Germany, in which a value of 21 mm Hg corresponded to 2 standard deviations above the mean IOP.^19^ This statistical concept of a so-called normal IOP range subsequently permeated clinical research reports, medical student and resident education, and even prior national referral guidelines.^20,21^ More recently, numerous studies have highlighted a continuous (rather than dichotomous) association between IOP and glaucoma risk—meaning that the risk of glaucoma increases with rising IOPs, even at levels below 22 mm Hg.^20,22,23,24,25,26^ Additionally, most patients with IOPs of 22 mm Hg or higher never go on to develop glaucoma, and over half of patients with glaucoma have IOPs consistently below 22 mm Hg.^21,27^ In other words, there is no clinical basis to support a true IOP cutoff of 22 mm Hg in glaucoma decision-making. In our assessment of multiple academic eye centers, IOP-lowering treatment was initiated at gradually higher rates with increasing IOPs, even at levels below the historical cutoff of 22 mm Hg. As such, clinicians generally seemed to align with the concept of IOP as a continuous risk factor for glaucoma in their clinical practice. However, we also found a disproportionate increase in both glaucoma treatment rate and treatment probability at IOPs of 22 mm Hg or higher compared with IOPs less than 22 mm Hg. Our findings suggest that clinicians may still be influenced by the historical IOP cutoff of 22 mm Hg rather than fully using IOP as a continuous variable when making their glaucoma treatment decisions.

Continuous variables abound in the context of clinical ophthalmology, ophthalmic research, and throughout other branches of medicine.^28,29,30^ In the field of glaucoma specifically, continuous variables, such as IOP, central corneal thickness, and cup-disc ratio, play essential roles in estimating disease severity and risk of progression.^2^ However, multiple studies have suggested that limitations in human cognition and memory make it difficult for clinicians to efficiently and accurately interpret continuous variables, particularly when faced with numerous types and sources of clinical data.^31,32^ This can lead clinicians to rely on heuristics or cognitive shortcuts (such as dichotomization and categorization) to simplify continuous variables into more comprehensible chunks of information.^28,29,30^ Although the traditional dichotomization of IOP into normal and abnormal categories is no longer considered clinically accurate for glaucoma risk, the ongoing influence of this historical IOP cutoff may represent a type of heuristic that clinicians use to navigate the complex task of synthesizing clinical data to make glaucoma treatment decisions.

The role of heuristics in medical decision-making is poorly understood but has been of growing interest in recent years.^33,34^ Heuristics can help to reduce physician cognitive load and maximize decision-making efficiency. However, these decisional shortcuts can also foster cognitive biases that impact clinical judgment.^31,33,34^ For instance, statisticians and behavioral economists have warned that dichotomization of continuous variables can lead to oversimplification of valuable clinical information and mischaracterization of patient disease.^28,29,35^ Our findings suggest that the historical concept of IOP dichotomization may still impact decision-making in glaucoma management to some degree, with clinicians being significantly more likely to initiate or escalate treatment at IOPs of 22 mm Hg or higher compared with IOPs of 21 mm Hg or lower. While it is important to understand the potential influence of cognitive biases on clinical decision-making, we emphasize that the clinicians in our study did not fully treat IOP as a dichotomous variable, as they still demonstrated increasing treatment rates at higher IOPs even below the historical cutoff of 22 mm Hg.

Improved clinical decision support tools could be useful for combating potential cognitive biases and helping clinicians more effectively use IOP as a continuous risk factor in their glaucoma management. Prior studies suggest that one of the most challenging aspects of patient care is having to process and interpret large amounts of longitudinal clinical information within short periods of time.^32,36,37^ Computer-based clinical decision support systems aim to alleviate this challenge by efficiently synthesizing clinical data to assist clinicians with patient care decisions.^38^ In ophthalmology, clinical decision support systems are already being developed to help clinicians process longitudinal visual field data and determine appropriate follow-up intervals for patients with glaucoma.^36,37,39^ We suggest that future clinical decision support systems aimed at processing and interpreting longitudinal IOP information may be useful to alleviate cognitive burden and help clinicians more effectively apply IOP as a continuous risk factor in clinical practice. We also recognize that while IOP is an important risk factor for glaucoma, many other clinical factors can influence the decision to initiate IOP-lowering therapy, and future clinical decision support tools would need to account for this complexity.

### Limitations

Our study had limitations. Given the retrospective and deidentified nature of the SOURCE data repository, we cannot know the exact reasoning of individual clinicians to initiate or escalate IOP-lowering treatment. Glaucoma management is a complex and nuanced endeavor, and while IOP is often a driving force in treatment decisions, it is rarely the sole influencing factor. Multiple other factors that are not consistently available within the SOURCE database—including patient race, family history, corneal hysteresis, target IOP, disease severity, patient health and life expectancy, and measures of disease progression, such as visual field results, optical coherence tomography findings, and optic disc hemorrhages—can influence treatment decision-making in glaucoma care. While our study evaluated initiation and escalation of treatment relative to IOP level, it is difficult to assess for de-escalation (reduction or cessation) of treatment from database information alone; to investigate the association between IOP level and treatment de-escalation, nondatabase retrospective or prospective clinical studies would be useful. Additionally, the SOURCE database is limited in that it does not consistently provide the specific tonometry device used or patient circumstances (such as anxiety level) during IOP measurements. It also does not provide clinician characteristics, such as clinician age, race, training history, or years of practice, which may also influence clinical decision-making. Finally, there is potential for selection bias in our study, as we focused specifically on academic centers within the SOURCE consortium. Our study did not include smaller, independent ophthalmology and optometry practices and therefore may not be representative of treatment patterns in those settings. Future studies evaluating clinical decision-making in private ophthalmology practices would be useful to further understand the influence of IOP level in such settings.

## Conclusions

Our study has potentially important implications not only for ophthalmologists, but also for medical practitioners as a whole. In the age of electronic health records, it is becoming increasingly common for clinicians to report unsustainable workloads and information overload, which can impact their ability to efficiently and accurately care for patients.^32^ We emphasize that while clinicians in our study appeared to largely use IOP as a continuous risk factor in their glaucoma treatment decisions, there may also be a lingering influence of the historical IOP cutoff on current clinical decision-making. It is essential for clinicians to recognize how our own cognitive limitations might lead to a reliance on decisional shortcuts that bias our decision-making. In the future, implementation of user-focused clinical decision support systems could help to reduce clinician reliance on heuristics and maximize clinical decision-making in ophthalmology and beyond.

## Notes

Supplement 1. eTable 1. List of National Drug Codes (NDC) and *Current Procedural Terminology* (*CPT*) Codes That Were Included as Glaucoma Treatments eTable 2. Interaction Effect Between Intraocular Pressure (IOP) and IOP Values Greater Than or Equal to the Indicator IOP (19, 20, 21, or 22 mm Hg)

Supplement 2. Nonauthor Collaborator Spreadsheet

Supplement 3. Data Sharing Statement
