# Supplementary material for: Influence of Intraocular Pressure on Clinical Decision-Making in Glaucoma Management
Source: JAMA Ophthalmol. 2026 Jan 8;144(2):167–73. doi: 10.1001/jamaophthalmol.2025.5593 (PMC12784266; doi:10.1001/jamaophthalmol.2025.5593)
Supplement: Supplement 1. — eTable 1. List of National Drug Codes (NDC) and Current Procedural Terminology (CPT) Codes That Were Included as Glaucoma Treatments eTable 2. Interaction Effect Between Intraocular Pressure (IOP) and IOP Values Greater Than or Equal to the Indicator IOP (19, 20, 21, or 22 mm Hg) [file jamaophthalmol-e255593-s001.pdf]

Supplemental Online Content

Polski A, Brintz BJ, Hess R, et al; the SOURCE Consortium. Influence of intraocular pressure on clinical decision-making in glaucoma management. *JAMA Ophthalmol*. Published online January 8, 2025. doi:10.1001/jamaophthalmol.2025.5593

eTable 1. List of National Drug Codes (NDC) and *Current Procedural Terminology* (*CPT*) Codes That Were Included as Glaucoma Treatments
eTable 2. Interaction Effect Between Intraocular Pressure (IOP) and IOP Values Greater Than or Equal to the Indicator IOP (19, 20, 21, or 22 mm Hg)

This supplemental material has been provided by the authors to give readers additional information about their work.

© 2025 Polski A et al; the SOURCE Consortium. *JAMA Ophthalmology*.

eTable 1. List of National Drug Codes (NDC) and *Current Procedural Terminology* (*CPT*) Codes That Were Included as Glaucoma Treatments

Category (Code type)	Variable	Code numbers
IOP-lowering drops (NDC)	Alpha agonists	0023-9177-05, 0023-9177-10, 0023-9177-15, 0023-9321-03, 0023-9321-05, 0023-9321-10, 0023-9321-15, 0065-0660-10, 0299-5980-00, 0299-5980-02, 0299-5980-30, 0299-5980-35, 0299-5980-45, 14445-400-05, 14445-400-10, 14445-400-15, 17478-715-10, 17478-715-11, 17478-715-12, 17478-716-10, 17478-716-11, 24208-411-05, 24208-411-10, 24208-411-15, 50090-1046-0, 50090-1800-0, 50090-4224-0, 61314-143-05, 61314-143-10, 61314-143-15, 61314-144-05, 61314-144-10, 61314-144-15, 61314-665-05, 61314-665-10, 70069-231-01, 70069-232-01, 70069-233-01, 0023-9211-03, 0023-9211-05, 0023-9211-10, 0023-9211-15, 0065-4147-25, 0065-4147-27, 0078-0904-38, 0078-0904-98

© 2025 Polski A et al; the SOURCE Consortium. *JAMA Ophthalmology*.

Beta blockers	0023-9211-03, 0023-9211-05, 0023-9211-10, 0023-9211-15, 0065-0246-10, 0065-0246-15, 0187-1496-05, 0187-1496-99, 0187-1498-25, 0378-0055-01, 0378-0221-01, 0378-0715-01, 10702-013-01, 10702-014-01, 13811-618-10, 13811-620-10, 17478-189-24, 17478-288-10, 17478-288-11, 17478-288-12, 17478-288-25, 17478-289-10, 17478-289-11, 17478-289-12, 17478-289-25, 17478-365-05, 17478-366-05, 17478-366-10, 17478-366-15, 17478-705-10, 17478-705-11, 17478-705-12, 17478-705-25, 24208-004-01, 24208-004-02, 24208-004-03, 24208-812-05, 24208-813-05, 24208-813-10, 24208-814-25, 24208-816-05, 24208-818-25, 24208-819-05, 24658-700-01, 24658-701-01, 42806-038-01, 42806-038-10 ,42806-039-01, 42806-039-10, 45865-121-01, 50090-0558-0, 50090-3441-0, 50090-5091-0, 50383-021-05, 50383-021-10, 50383-021-15, 60429-753-01, 60429-754-01, 60505-1005-1, 60505-1005-4, 60758-801-05, 60758-801-10, 60758-802-05, 60758-802-10, 61314-224-05, 61314-224-25, 61314-225-05, 61314-225-25, 61314-226-05, 61314-226-10, 61314-226-15, 61314-227-05, 61314-227-10, 61314-227-15, 61314-245-01, 61314-245-02, 61314-245-03, 62332-545-05, 62332-546-05, 63629-7167-1, 63629-7167-2, 63629-7167-3, 63629-7167-4, 64980-513-01, 64980-513-05, 64980-513-15, 64980-514-01, 64980-514-05, 64980-514-15, 67877-229-11, 67877-229-15, 67877-229-55, 68682-045-25, 68682-045-50, 68682-812-05, 68682-813-05, 68682-813-10, 70518-2353-0, 76478-001-05, 76478-001-10, 76478-001-15, 76478-002-05, 76478-002-10, 76478-002-12, 76478-002-15, 76519-1163-0, 0527-1763-73, 17478-514-11, 17478-604-15, 17478-604-30, 17478-604-90, 17478-605-10, 24208-486-05, 24208-486-10, 42571-147-26, 50090-1247-0, 50383-233-05, 50383-233-10, 50383-261-61, 50383-261-91, 60429-115-10, 61314-030-01, 61314-030-02, 65862-947-18, 65862-947-60, 69315-305-05, 69315-305-10, 70069-051-12

© 2025 Polski A et al; the SOURCE Consortium. *JAMA Ophthalmology*.

	Carbonic anhydrase inhibitors	0006-3519-36, 0065-0275-10, 0065-0275-15, 0065-0275-25, 0998-0203-15, 0998-0204-15, 0998-0206-15, 17478-223-12, 17478-224-12, 17478-226-12, 24208-485-10, 42571-141-26, 50090-1246-0, 50090-5280-0, 50383-232-05, 50383-232-10, 60219-1745-8, 60219-1746-8, 60219-1747-8, 60429-114-10, 61314-019-10, 61314-203-15, 61314-204-15, 61314-206-15, 62332-519-10, 69238-1745-8, 69238-1746-8, 69238-1747-8, 69315-304-05, 69315-304-10, 70069-181-01, 70069-191-01, 70069-201-01, 0527-1763-73, 17478-514-11, 17478-604-15, 17478-604-30, 17478-604-90, 17478-605-10, 24208-486-05, 24208-486-10, 42571-147-26, 50090-1247-0, 50383-233-05, 50383-233-10, 50383-261-61, 50383-261-91, 60429-115-10, 61314-030-01, 61314-030-02, 65862-947-18, 65862-947-60, 69315-305-05, 69315-305-10, 70069-051-12, 0065-4147-25, 0065-4147-27, 0078-0904-38, 0078-0904-98
	Prostaglandin analogues	0013-8303-04, 0023-3205-02, 0023-3205-03, 0023-3205-05, 0023-3205-08, 0065-0260-02, 0065-0260-05, 0065-0260-25, 0078-0946-25, 0078-0946-40, 0078-0946-98, 0378-9651-32, 0378-9651-50, 0781-6185-56, 0781-6185-75, 0781-6206-75, 0781-6206-93, 13985-610-02, 17478-609-10, 17478-609-30, 17478-609-90, 17478-625-12, 24208-463-25, 47335-317-90, 47335-317-92, 47335-317-94, 47335-317-98, 50383-908-02, 50383-908-05, 50383-908-07, 50383-912-03, 50383-912-05, 59762-0333-2, 60505-0583-1, 60505-0583-4, 60505-0583-5, 60505-0593-1, 60505-0593-4, 61314-547-01, 61314-547-03, 61919-112-25, 62332-507-05, 62332-507-25, 62332-510-05, 62332-510-25, 62332-511-03, 62332-511-05, 64980-516-25, 65862-872-25, 68071-4612-2, 68071-4650-2, 68071-4893-2, 68083-295-01, 68083-296-01, 68180-429-01, 68180-429-02, 68180-429-03, 70069-401-01, 70069-402-01, 70069-403-01, 70069-421-01, 70069-421-03, 71205-154-25, 72266-139-01, 72266-140-01, 24208-504-01, 24208-504-02, 24208-504-05, 24208-504-06, 70727-529-25, 70727-529-99
	Nitric oxide donators	24208-504-01, 24208-504-02, 24208-504-05, 24208-504-06
	Rho kinase inhibitors	70727-497-25, 70727-497-99, 70727-529-25, 70727-529-99

© 2025 Polski A et al; the SOURCE Consortium. *JAMA Ophthalmology*.

Glaucoma surgeries and laser procedures (CPT)	Incisional glaucoma shunt surgery	1261, 1264, 1265, 1267, C1783, L8612, 66160, 66170, 66172, 66179, 66180, 66184, 66185
	MIGS drainage devices	0191T, 0253T, 0376T, 0449T, 0450T, 0474T, 66183, 66989, 66991
	Other MIGS without drainage devices	65820, 65850, 66174, 66175
	Laser glaucoma procedures	65855, 66710, 66711

© 2025 Polski A et al; the SOURCE Consortium. *JAMA Ophthalmology*.

eTable 2. Interaction Effect Between Intraocular Pressure (IOP) and IOP Values Greater

Than or Equal to the Indicator IOP (19, 20, 21, or 22 mm Hg)

Indicator IOP	Interaction Estimate	95% CI	p-value
**19 mm Hg**	0.0035	0.0025, 0.0045	<0.001
**20 mm Hg**	0.0056	0.0047, 0.0065	<0.001
**21 mm Hg**	0.0073	0.0064, 0.0082	<0.001
**22 mm Hg**	0.0090	0.0081, 0.0099	<0.001

The interaction estimate represents the change in slope from IOP values below the indicator IOP compared to IOP values greater than or equal to the indicator IOP and demonstrates the largest slope change at an indicator IOP of 22 mm Hg.

© 2025 Polski A et al; the SOURCE Consortium. *JAMA Ophthalmology*.
