# Supplementary material for: Influence of Intraocular Pressure on Clinical Decision-Making in Glaucoma Management
Source: JAMA Ophthalmol. 2026 Jan 8;144(2):167–73. doi: 10.1001/jamaophthalmol.2025.5593 (PMC12784266; doi:10.1001/jamaophthalmol.2025.5593)
Supplement: Supplement 2. — Nonauthor Collaborator Spreadsheet [file jamaophthalmol-e255593-s002.pdf]

Supplemental Online Content: Nonauthor Collaborators
*First name, last name, and suffix (if applicable) are required and will appear in PubMed.

*Group Name(s): Sight Outcomes Research Collaborative (SOURCE) Consortium							
***First Name and Middle Initial(s)**	***Last Name**	***Suffix (eg, Jr, III)**	Academic Degrees	Institution	Location (city, state/province, country)	Role or Contribution, eg, chair, principal investigator	Group (if more than 1 Group listed in the byline) and/or Subgroup (eg, Steering Committee)
Sejal	Amin		MD	Henry Ford Health System	Detroit, MI	Site PI	
Paul A.	Edwards		MD	Henry Ford Health System	Detroit, MI	Site PI	
Divya	Srikumaran		MD	Johns Hopkins University	Baltimore, MD	Site PI	
Fasika	Woreta		MD	Johns Hopkins University	Baltimore, MD	Site PI	
Jeffrey S	Schultz		MD	Montefiore Medical Center	Bronx, NY	Site PI	
Anurag	Shrivastava		MD	Montefiore Medical Center	Bronx, NY	Site PI	
Baseer	Ahmad		MD	Medical College of Wisconsin	Milwaukee, WI	Site PI	
Louis R	Pasquale		MD	Mount Sinai School of Medicine	New York, NY	Site PI	
Paul J	Bryar		MD	Northwestern University	Chicago, IL	Site PI	
Dustin D	French		PhD	Northwestern University	Chicago, IL	Site PI	
Michelle	Hribar		PhD	Oregon Health Sciences University	Portland, OR	Site PI	
Merina	Thomas		MD	Oregon Health Sciences University	Portland, OR	Site PI	
Brian L	Vanderbeek		MD	University of Pennsylvania	Philadephia, PA	Site PI	
Suzann	Pershing		MD	Stanford University	Palo Alto, CA	Site PI	
Sophia Y	Wang		MD	Stanford University	Palo Alto, CA	Site PI	
Preethi	Ganapathy		MD, PhD	SUNY Upstate	Sryacuse, NY	Site PI	
Michael	Deiner		PhD	University of California, San Francisco	San Francisco, CA	Site PI	
Catherine	Sun		MD	University of California, San Francisco	San Francisco, CA	Site PI	
Jennifer	Patnaik		PhD	University of Colorado	Denver, CO	Site PI	
Prem	Subramanian		MD	University of Colorado	Denver, CO	Site PI	
Saleha	Munir		MD	University of Maryland	Baltimore, MD	Site PI	
Wuqaas	Munir		MD	University of Maryland	Baltimore, MD	Site PI	
Joshua D	Stein		MD, MS	University of Michigan	Ann Arbor, MI	Site PI; Chief Data Officer	
Lindsey	DeLott		MD	University of Michigan	Ann Arbor, MI	Site PI	
Rajeev	Ramachandran		MD	University of Rochester	Rochester, NY	Site PI	
Robert	Feldman		MD	University of Texas, Houston	Houston, TX	Site PI	
Brian C	Stagg		MD, MS	University of Utah	Salt Lake City, UT	Site PI	
Barbara	Wirostko		MD	University of Utah	Salt Lake City, UT	Site PI	
Brian	McMillian		MD	West Virginia University	Morgantown, WV	Site PI	
Arsham	Sheybani		MD	Washington University	St Louis, MO	Site PI	
Soshian	Sarrapour		MD	Yale University	New Haven, CT	Site PI	
Kristen	Harris-Nwanyanwu		MD	Yale University	New Haven, CT	Site PI	
Arjun	Dirghangi		MD	University of Virginia	Charlottesville, VA	Site PI	
Chris	Andrews		PhD	University of Michigan	Ann Arbor, MI	Lead Statistician	
