# Supplementary material for: Influence of Intraocular Pressure on Clinical Decision-Making in Glaucoma Management
Source: JAMA Ophthalmol. 2026 Jan 8;144(2):167–73. doi: 10.1001/jamaophthalmol.2025.5593 (PMC12784266; doi:10.1001/jamaophthalmol.2025.5593)
Supplement: Supplement 3. — Data Sharing Statement [file jamaophthalmol-e255593-s003.pdf]

Data Sharing Statement

Polski. Influence of Intraocular Pressure on Clinical Decision-Making in Glaucoma Management. *JAMA Ophthalmol*. Published January 08, 2026.
doi:10.1001/jamaophthalmol.2025.5593

Data
Data available: No
